# Mycophenolate mofetil prevents the delayed T cell response after pilocarpine-induced status epilepticus in mice

**DOI:** 10.1371/journal.pone.0187330

**Published:** 2017-11-28

**Authors:** Anne-Marie Neumann, Julia Abele, Timo Kirschstein, Robby Engelmann, Tina Sellmann, Rüdiger Köhling, Brigitte Müller-Hilke

**Affiliations:** 1 Institute of Immunology, University of Rostock, Rostock, Germany; 2 Oscar Langendorff Institute of Physiology, University of Rostock, Rostock, Germany; University of Modena and Reggio Emilia, ITALY

## Abstract

Growing clinical and laboratory evidence corroborates a role for the immune system in the pathophysiology of epilepsy. In order to delineate the immune response following pilocarpine-induced status epilepticus (SE) in the mouse, we monitored the kinetics of leukocyte presence in the hippocampus over the period of four weeks. SE was induced following a ramping protocol of pilocarpine injection into 4–5 weeks old C57BL/6 mice. Brains were removed at days 1–4, 14 or 28 after SE, and the hippocampi were analyzed via flow cytometry, via quantitative reverse transcriptase PCR (qRT-PCR) and via immunohistochemistry. Epileptogenesis was confirmed by Timm staining of mossy fiber sprouting in the inner molecular layer of the dentate gyrus. The flow cytometry data revealed a biphasic immune response following pilocarpine-induced SE with a transient increase in activated CD11b^+^ and F4/80^+^ macrophages within the first four days replaced by an increase in CD3^+^ T-lymphocytes around day 28. This delayed T cell response was confirmed via qRT-PCR and via immunohistochemistry. In addition, qRT-PCR data could show that the delayed T cell response was associated with an increased CD8/CD4 ratio indicating a cytotoxic T cell response after SE. Intriguingly, early intervention with mycophenolate mofetil administration on days 0–3 after SE prevented this delayed T cell response. These results show an orchestrated immunological sequela and provide evidence that the delayed T cell response is sensitive to early immunomodulatory intervention.

## Introduction

There is currently a growing body of evidence linking brain inflammation to the pathophysiology of human epilepsy. Common infections and autoimmune diseases are accepted to cause epileptic encephalopathies as are sterile inflammatory reactions following brain injuries like neurotrauma, stroke, febrile convulsion and status epilepticus [1,2]. However, the role of individual immune cells and exact mechanisms are only slowly being unraveled. For example, the activation of innate immune cells of the microglia/macrophage lineage has been associated with a sustained pro-inflammatory chemokine and cytokine response that affects the permeability of the blood brain barrier [3,4] and precedes the establishment of recurrent seizures [5–7]. As of yet, little is known about the involvement of the adaptive immune system. In contrast, Rasmussen's encephalitis has been associated with autoantibodies directed against the glutamate receptor subunit 3 [8] and cytotoxic CD8 cells have been suggested to mediate the attack on neurons and astrocytes in this autoimmune disease [9,10]. In order to identify in depth the immunological mechanisms involved and to delineate the kinetics leading to the development of epilepsy, animal models are indispensable.

Pilocarpine and kainate induced status epilepticus in rodents are both models leading to temporal lobe epilepsy [11]. CD3^+^ T-cell infiltration into the brain parenchyma has been shown for mice following kainate-induced status epilepticus as well as for human tissue from temporal lobe epilepsy (TLE) patients undergoing epilepsy surgery. In contrast, B220^+^ B-cells were not observed in these specimens [12].

The present study was performed to investigate the immunological sequelae following pilocarpine-induced status epilepticus in mice [13]. In the past, systemic pilocarpine administration in mice was often compromised by a high lethality and a low rate of mice with fully developed SE [14–17]. Recently, a ramping protocol with repeated injections of low doses of pilocarpine was established and successfully reduced these complications [18–20]. We adapted this protocol in order to study the immune response after pilocarpine-induced SE. The aim of this study was to explore the early immune response following SE [12,21] and to investigate later immunological sequelae in the pilocarpine-induced epilepsy model. In addition, we studied the effects of an early pharmacological intervention with mycophenolate mofetil.

## Materials and methods

### Pilocarpine-induced status epilepticus

A prolonged status epilepticus (SE) was induced using a ramping pilocarpine protocol [18–20] in male 28-to-34-day-old C57BL/6 mice (Charles River, Sulzfeld, Germany). All procedures were performed according to national and international guidelines on the ethical use of animals (European Council Directive 86/609/EEC, approval of local authority, State Office for Agriculture, Food Safety and Fisheries Mecklenburg-Vorpommern, Germany; LALLF M-V/TSD/7221.3–1.1-013/10 and LALLF M-V/TSD/7221.3–1.1-047/12). Peripheral cholinergic effects were reduced by pre-application of methyl-scopolamine nitrate (Sigma-Aldrich, 1 mg/kg, i.p.) 30 min prior to pilocarpine treatment. Then, pilocarpine hydrochloride (Sigma-Aldrich, 100 mg/kg, i.p.) or saline as control was administered. The pilocarpine injection (100 mg/kg, i.p.) was repeated every 20 min until the animals developed SE (up to 800 mg/kg; on average 457±113 mg/kg, mean ± SD). During this procedure, all animals were carefully observed in individual cages. Control animals received saline instead of pilocarpine. Ten percent of animals were randomized to get saline instead of pilocarpine as control, but due to overt behavioral seizure activity the experimenter could not be blinded to whether the animal was injected with pilocarpine or with saline. The onset of SE was determined when an animal had a stage 4 or 5 seizure [22], which was followed by continuous epileptic motor activity. After 90 min of SE duration, all animals received a 100 μl bolus injection of diazepam solution (Ratiopharm, Ulm, Germany, 5 mg/ml, i.p.). Occasionally, diazepam had to be re-injected in order to stop seizure activity (up to 200 μl altogether). Using this ramping pilocarpine protocol, 54% of animals developed and survived status epilepticus. At last, all animals were given 100 μl of 0.9% saline solution and kept in separate cages.

### Mossy fiber sprouting

Epileptogenesis is associated with characteristic mossy fiber sprouting in the inner molecular layer of the dentate gyrus [14], which can be demonstrated by the use of the Timm stain [23]. Therefore, we have performed Timm stains with brains from 31 pilocarpine-treated mice at 2–5 months after SE and 10 control animals as previously described [24]. Briefly, animals were decapitated in deep anesthesia with diethyl ether (Merck Biosciences). The brains were cut into hemispheres and incubated in Na_2_S-containing solution (1.17% Na_2_S in 0.15 M phosphate buffered saline, PBS) at 4°C for at least 24 hours in order to stain the zinc-positive mossy fibers in the epileptic hippocampus. Subsequently, brains were immersed in 0.3% glutaraldehyde (dissolved in PBS) at 4°C for at least a few days, and cryo-protected in 30% sucrose solution. Cryostat sections (20 μm) were stained with 0.1% AgNO_3_-containing developing solution (1.7% hydrochinone in citrate-buffer) for 20–40 min in the dark, and transferred into stopping solution (1% Na_2_S_2_O_3_ solution). Nuclei were counterstained with toluidine blue (0.25% toluidine blue in 1% borax solution).

### Flow cytometry

For flow cytometric analyses, animals were decapitated in deep anesthesia with diethyl ether at different time points following SE (1–4 days, 14 days, 28 days), and the brains were quickly removed. The hippocampus formations of both hemispheres were carefully prepared under the dissection microscope (Leica), and directly immersed into ice-cold Dulbecco’s modified eagle medium (Invitrogen). In preliminary experiments, we have found out that pooling the hippocampi from 2–4 animals was required to yield sufficient cell counts for flow cytometric analyses. On average, overall cell counts of hippocampal tissues were not significantly different between pilocarpine-treated (990±95/μl, n = 38 samples from 93 animals) and control animals (1071±119/μl, n = 30 samples from 77 animals).

The tissue specimens were digested with collagenase A (1 mg/ml, Roche Diagnostics) and DNase type IV (0.1 mg/ml, Sigma) for 30 min at 37°C. The digested cell suspensions were filtered (70 μm cell filter) and centrifuged (1200 rpm at 4°C for 10 min) and subsequently loaded onto a percoll gradient (Sigma) in order to isolate hippocampal immune cells (personal communication Jan Sodenkamp). The resuspended isolated cells were stained with fluorochrome-conjugated antibodies against CD45 (FITC anti-mouse CD45, clone 30-F11, RRID: AB_312972), CD11b (PerCP anti-mouse/human CD11b, clone M1/70, RRID: AB_2129374), CD3 (PerCP anti-mouse CD3ɛ, clone 145-2C11, RRID: AB_893319), B220 (APC anti-mouse/human CD45R/B220, cloneRA3-6B2, RRID: AB_312997), Nk1.1 (APC anti-mouse NK-1.1, clone PK136, RRID: AB_313396) and F4/80 (APC anti-mouse F4/80, cloneBM8, RRID: AB_893481) in four different panels. All antibodies were purchased from Biolegend (San Diego, CA). The samples were analyzed on a BD FACSCaliburflow cytometer (BectonDickinson, BD) and subsequently quantified using the Cell Quest (BD) or WinMDI2.9 software.

### Quantitative RT-PCR

For RNA isolation, hippocampal tissues were freshly prepared as described above (decapitation in deep anesthesia with diethyl ether) and further processed using RNeasy Plus Mini Kits (Qiagen, Germany) according to the manuals. The isolated RNA was transcribed into cDNA by High Capacity cDNA Reverse Transcription Kit (Applied Biosystems, Germany). The expression of the following genes was quantified by TaqMan Gene Expression Assays according to the manuals. Gene specific probes for GAPDH, Emr1 (F4/80, macrophages), CD3g (T-lymphocytes), Ptprc (CD45, leukocytes), CD4 (T helper cells) and CD8 (cytotoxic T cells) were purchased from Applied Biosystems. The ΔCT was determined by comparing the expression of the gene of interest to Ptprc, the ΔΔCT was determined by comparing MMF and/or pilocarpine treated animals to non-treated controls. The relative expression differences were defined as 2^-^Δ^CT^ and 2^-ΔΔCT^, respectively.

### Mycophenolate mofetil administration

A subset of animals (6 pilocarpine-treated and 8 control animals) received a bolus injection of the purine antagonist mycophenolate mofetil (20 mg/kg i.p.) directly after the pilocarpine-induced SE (day 0). The administration was repeated four times with daily injections until day 3 after SE). Mycophenolate mofetil was well tolerated, and this intervention was approved by the local authority (LALLF M-V/TSD/7221.3–1.1-047/12). The hippocampi of all animals treated with mycophenolate mofetil were prepared on day 28 after SE and used for qRT-PCR.

### Immunohistochemistry

At one month after pilocarpine induced SE, 6 pilocarpine-treated and 4 control mice were i.p. treated with 75 μl of a 15% xylazine (Rompun®, Bayer) and 85% ketamine (Ketanest® S, Pfizer) mix prior to vascular perfusion. Then, animals were decapitated and whole brains were prepared and incubated with 3.7% paraformaldehyde (commercial 37% PFA solution, Roth CP10.3, Karlsruhe, Germany; diluted to the final concentration in phosphate-buffered saline, pH adjusted to 7.4) for at least 24 hours before they were transferred into a 30% sucrose solution. Cryostat sections (10 μm) were treated for 5 minutes using 10% methanol and 7% H_2_O_2_ in Tris buffer followed by 1 hour of blocking (10% bovine serum albumin, 1.5% DL-lysine and ~50 μl Triton X-100 in Tris buffer for 4 sections). Incubation with an anti-CD3 antibody (abcam ab5690) was performed over night at room temperature followed by an Alexa-488-conjugated secondary antibody (Invitrogen A11034) for 90 minutes, again at room temperature. Carrier solution for the antibodies was Tris buffer with 2% normal sheep serum. The sections were covered with ProLong® Gold antifade reagent with DAPI (Invitrogen) and evaluated using the Leica DMI6000 B microscope and LAS AF software.

### Data processing and statistical analysis

For data processing (mossy fiber sprouting, flow cytometry, quantitative RT-PCR), the experimenter was blinded to which the animal was allocated (pilocarpine-treated, control). All data are expressed as individual data points and the mean value (as horizontal lines). Data were first analyzed for normal distribution, statistical significance was tested using Student’s t-test or χ^2^test for normally distributed data, and otherwise Mann-Whitney U-test as indicated. The level of significance is indicated by asterisks (*P<0.05, **P<0.01).

## Results

**Fig 1:**
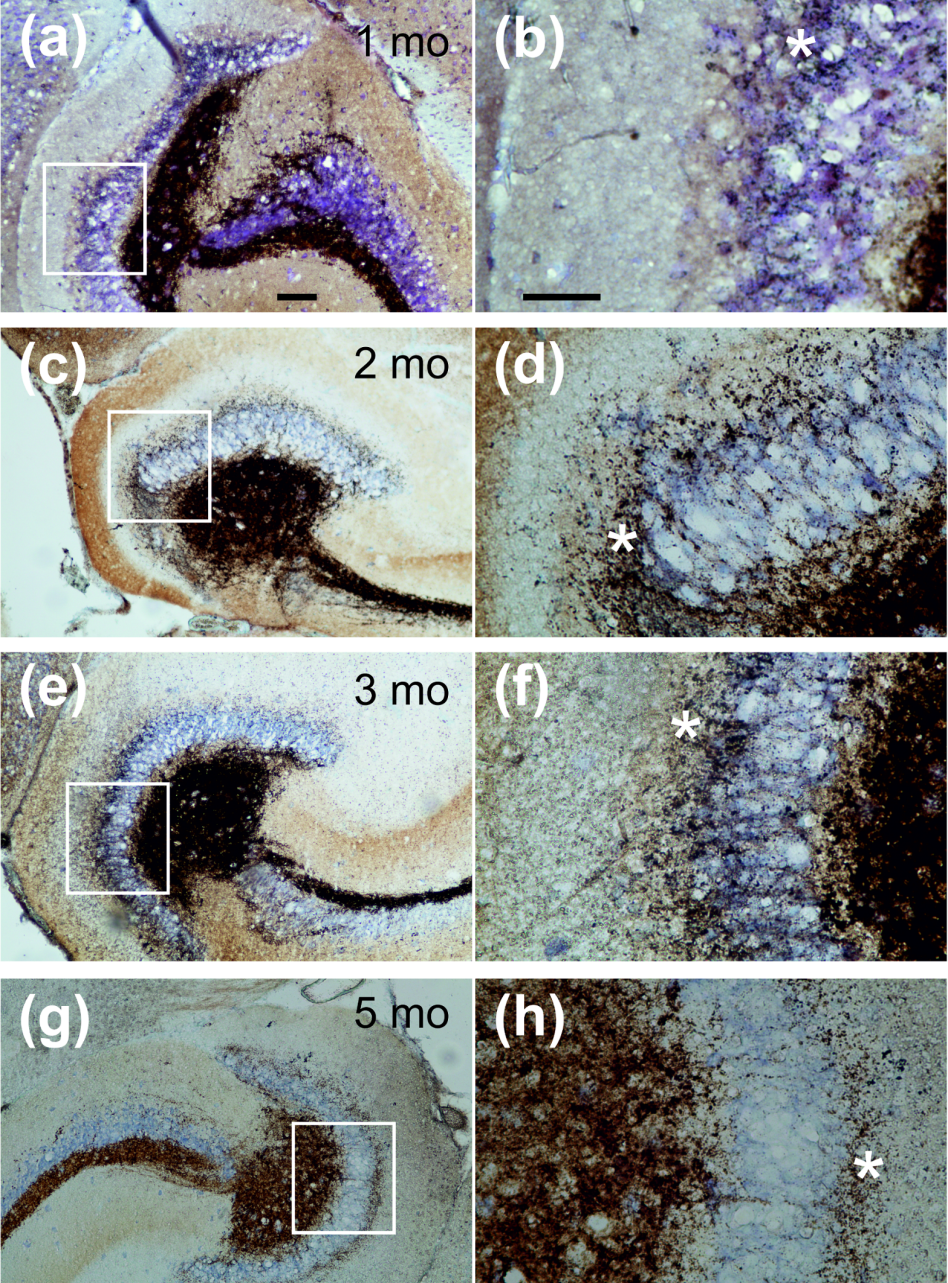
Mossy fiber sprouting in the inner molecular layer of the dentate gyrus. **(a,c,e,g)** Micrographs showing the dentate gyrus with increasing mossy fiber sprouting in the inner molecular layer at 1 month (a), 2 months (c), 3 months (e), and 5 months (g) after SE. The scale bar indicates 100 μm, magnification 100x. **(b,d,f,h)** Micrographs at higher magnification (400x, scale bar indicates 50 μm) taken at the white box indicated in panels (a,c,e,g). Note the dark brown spots within the inner molecular layer indicating zink-positive mossy fiber sprouting (indicated by white asterisk).

The aim of the present study was to characterize the immune response of the murine brain following pilocarpine-induced status epilepticus (SE). Timm staining was performed in order to demonstrate the typical mossy fiber sprouting in the inner molecular layer confirming epileptogenesis following pilocarpine-induced SE in mice (Fig 1A–1H). Mossy fiber sprouting is discernible after one month after SE (Fig 1A and 1B), but gets more pronounced with increasing time after SE (Fig 1C–1H). This indicates that epileptogenesis takes place within the first month after SE, but may aggravate during the course of the disease.

**Fig 2:**
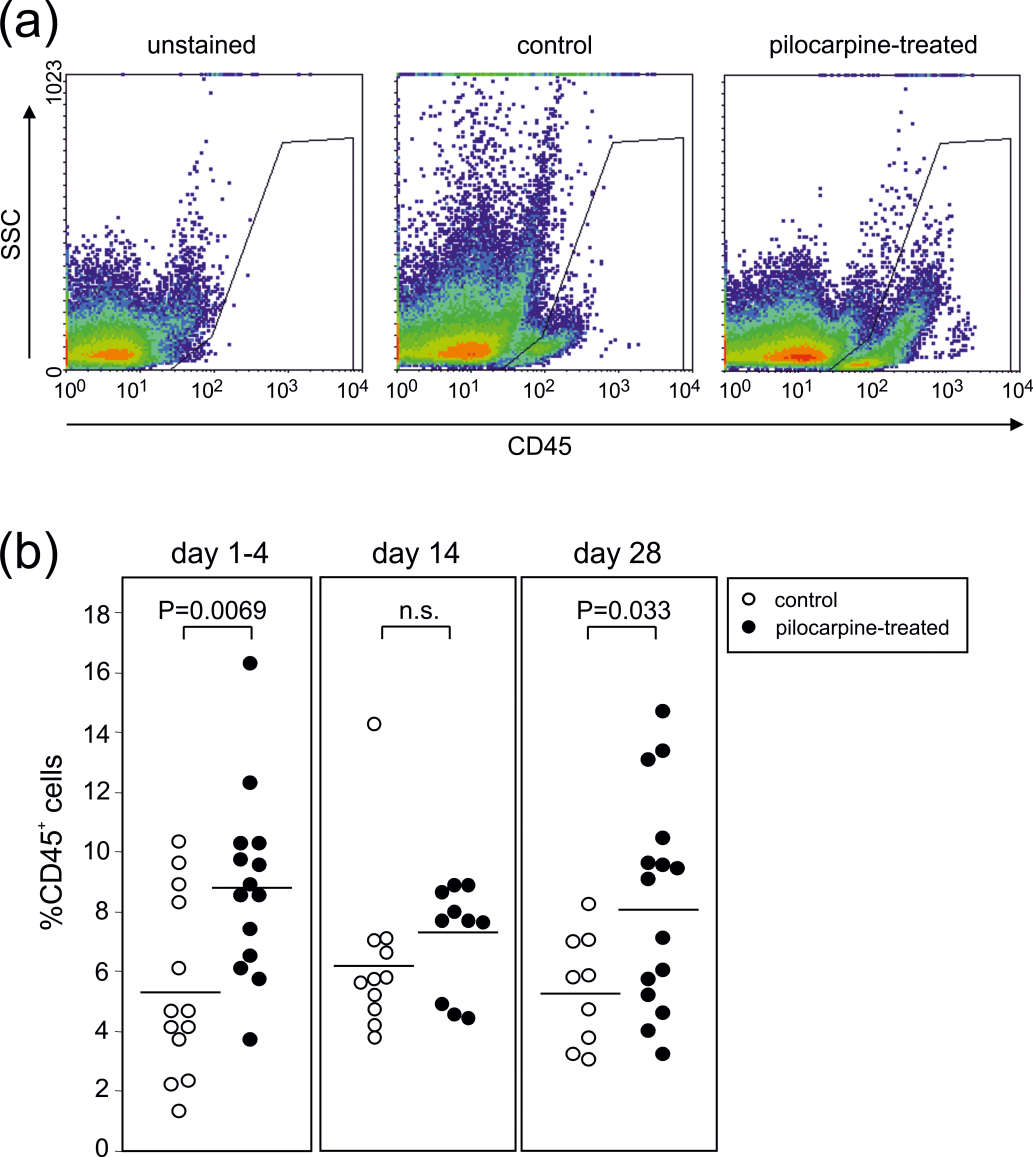
Biphasic immune response following pilocarpine-induced SE in the murine hippocampus. **(a)** Sideward scatter (SSC) versus CD45 analysis of control animals and animals on day 2 post SE. The gates describe the CD45^+^ leukocytes. **(b)** Percentages of CD45^+^ leukocytes found in the hippocampi of either control or pilocarpine treated animals on days 1–4, 14 and 28 post SE. Each symbol represents pooled hippocampal cells of 2–4 mice. Horizontal lines indicate means and P-values result from unpaired t-tests.

The immune response of the murine brain following pilocarpine-induced SE was assessed via flow cytometric analyses of hippocampus cell suspensions on days 1 to 4, 14 and 28 after SE. On days 1–4 post SE, we found CD45^+^ cells significantly enhanced in the hippocampus samples obtained from pilocarpine-treated mice (8.8±0.6%, n = 14 samples from 26 animals) in comparison to the controls (5.4±0.8%, n = 13 samples from 34 animals; P<0.01, t-test, Fig 2). Interestingly, this rise in CD45^+^ cells among pilocarpine-treated animals was no longer observed on day 14, but re-emerged on day 28 (pilocarpine-treated: 8.3±0.7%, n = 15 samples from 30 animals; control: 5.4±0.6%, n = 9 samples from 38 animals; P<0.05, t-test).

**Fig 3:**
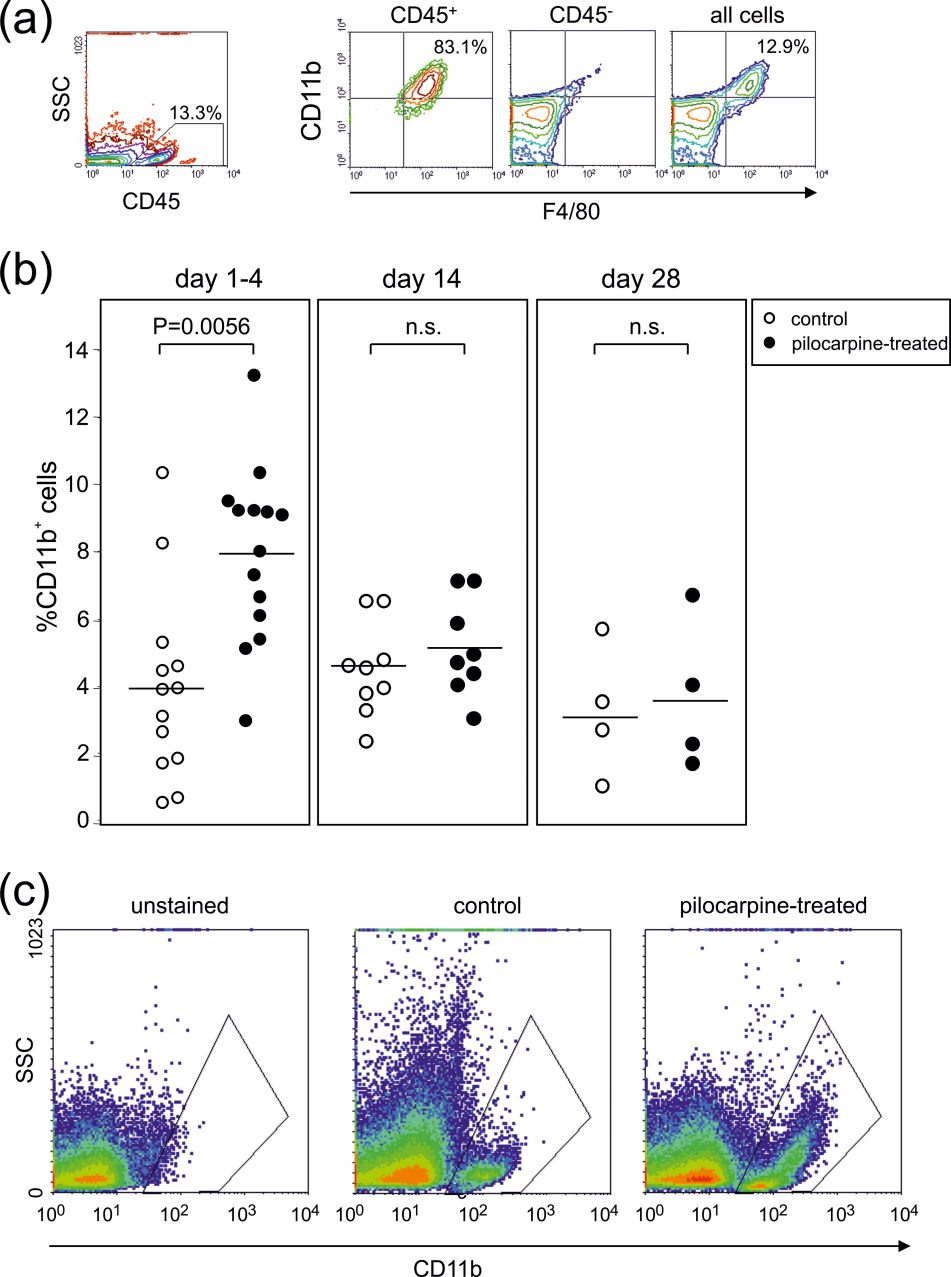
The early phase of the hippocampal immune response following pilocarpine-induced SE correlates with an increase in activated microglia. **(a)** The left panel depicts a sideward scatter versus CD45 analysis of hippocampus cells on day 4 post SE. The panels on the right depict the CD11b versus F4/80 staining gating on either CD45^+^, CD45^-^ or ungated cells. Numbers indicate percentages in the respective quadrants and gates. **(b)** Summary of percentages of CD11b^+^ leukocytes found in the hippocampi of either control or pilocarpine treated animals on days 1–4, 14 and 28 post SE. Each symbol represents pooled hippocampal cells of 2–4 mice. Horizontal lines indicate means and P-values result from unpaired t-tests. **(c)** Sideward scatter versus CD11b analysis of control animals and animals on day 2 post SE. The gates indicate the CD11b^+^ leukocytes.

To characterize the nature of these immune cells in more detail and to differentiate innate from adaptive responses, we introduced markers for the myeloid and lymphoid lineages, respectively. We thus found that on day 4, a mean of 90.1±1.7% of the leukocytes present in the hippocampus were positively stained for CD11b. Of these, a mean of 72.2±4.6% stained positive for both, CD11b and F4/80, indicating tissue resident microglia (Fig 3A). The very right panel gives an exemplary percentage of CD11b^+^/F4/80^+^ microglial cells among all hippocampal cells.

Fig 3B shows the percentages of CD11b^+^/F4/80^+^ microglial cells among all hippocampal cells on days 1–4, 14 and 28 after SE. Interestingly, the early rise in leukocytes on days 1–4 post SE (shown in Fig 2B) correlated with a significant increase in the CD11b^+^/F4/80^+^ microglial cells in the hippocampus (pilocarpine-treated: 7.9±0.8%, n = 14 samples from 26 animals; control: 4.2±0.9%, n = 13 samples from 34 animals; P<0.01, t-test). This increase was transient and no longer observed on days 14 and 28 (Fig 3B). Moreover, this early increase was accompanied by a highly granular phenotype of the CD11b^+^ cells supporting activation of the microglia (Fig 3C).

**Fig 4:**
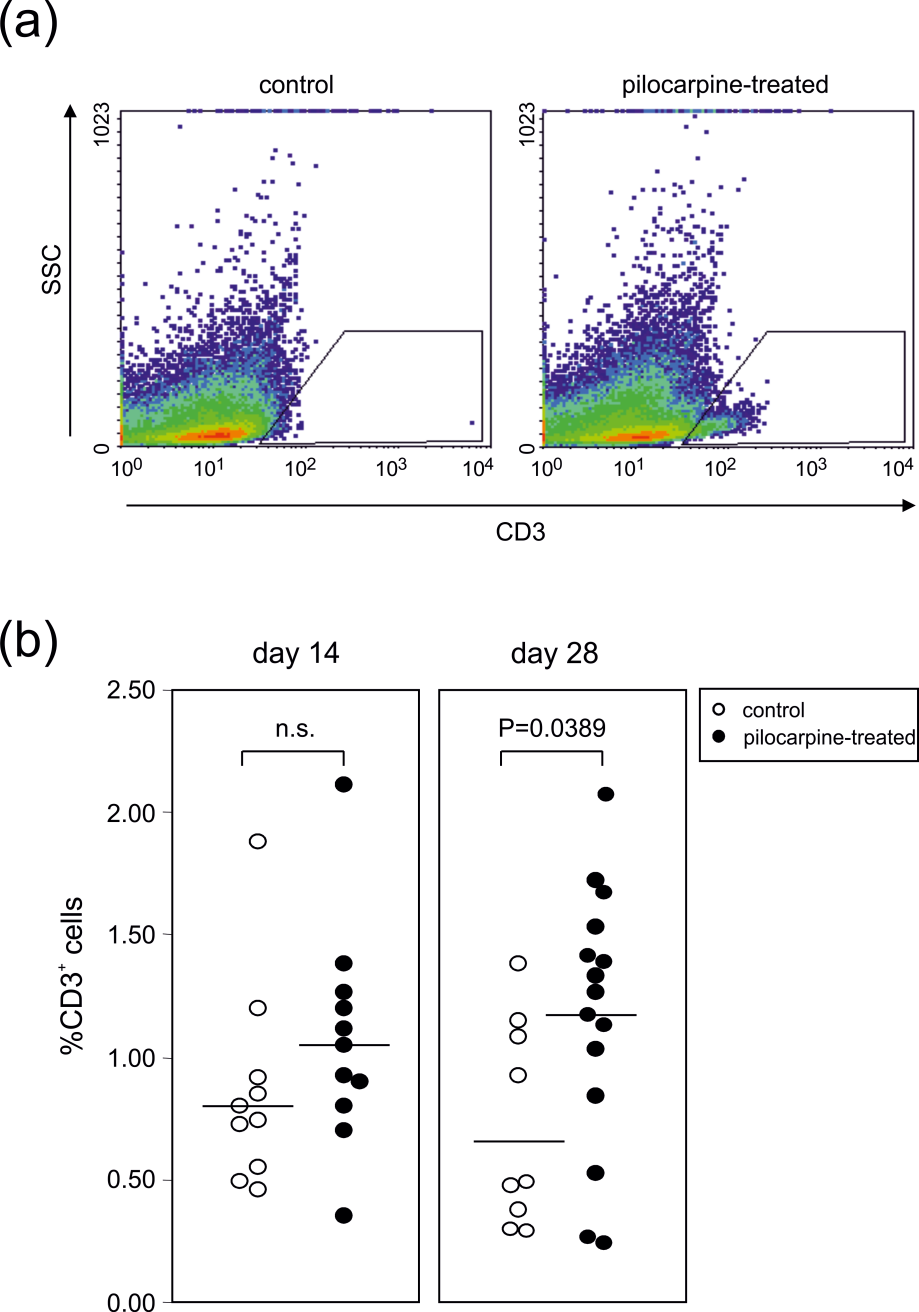
The late phase of the hippocampal immune response following pilocarpine-induced SE correlates with an increase in CD3^+^ T cells. **(a)** Sideward scatter versus CD3 analysis of control animals and animals on day 28 post SE. The gates indicate the CD3+ leukocytes. **(b)** Percentages of CD3^+^ leukocytes found in the hippocampi of either control or pilocarpine treated animals on days 14 and 28 post SE. Each symbol represents pooled hippocampal cells of 2–4 mice. Horizontal lines indicate means and P-values result from unpaired t-tests.

Importantly, days 14 and 28 revealed an increase in CD3^+^ T cells (compared to <0.1% on day 1–4), which reached significance at day 28 (pilocarpine-treated: 1.17±0.14%, n = 15 samples from 30 animals; control: 0.71±0.14%, n = 9 samples from 22 animals; P<0.05, t-test, Fig 4). This late rise in T cells correlated with the rise in leukocytes on day 28 post SE (shown in Fig 2B). We did not observe any pilocarpine-induced changes in the percentages of B220^+^ B-lymphocytes and NK1.1^+^ NK cells, neither on days 1–4 nor on days 14 or 28 post SE.

**Fig 5:**
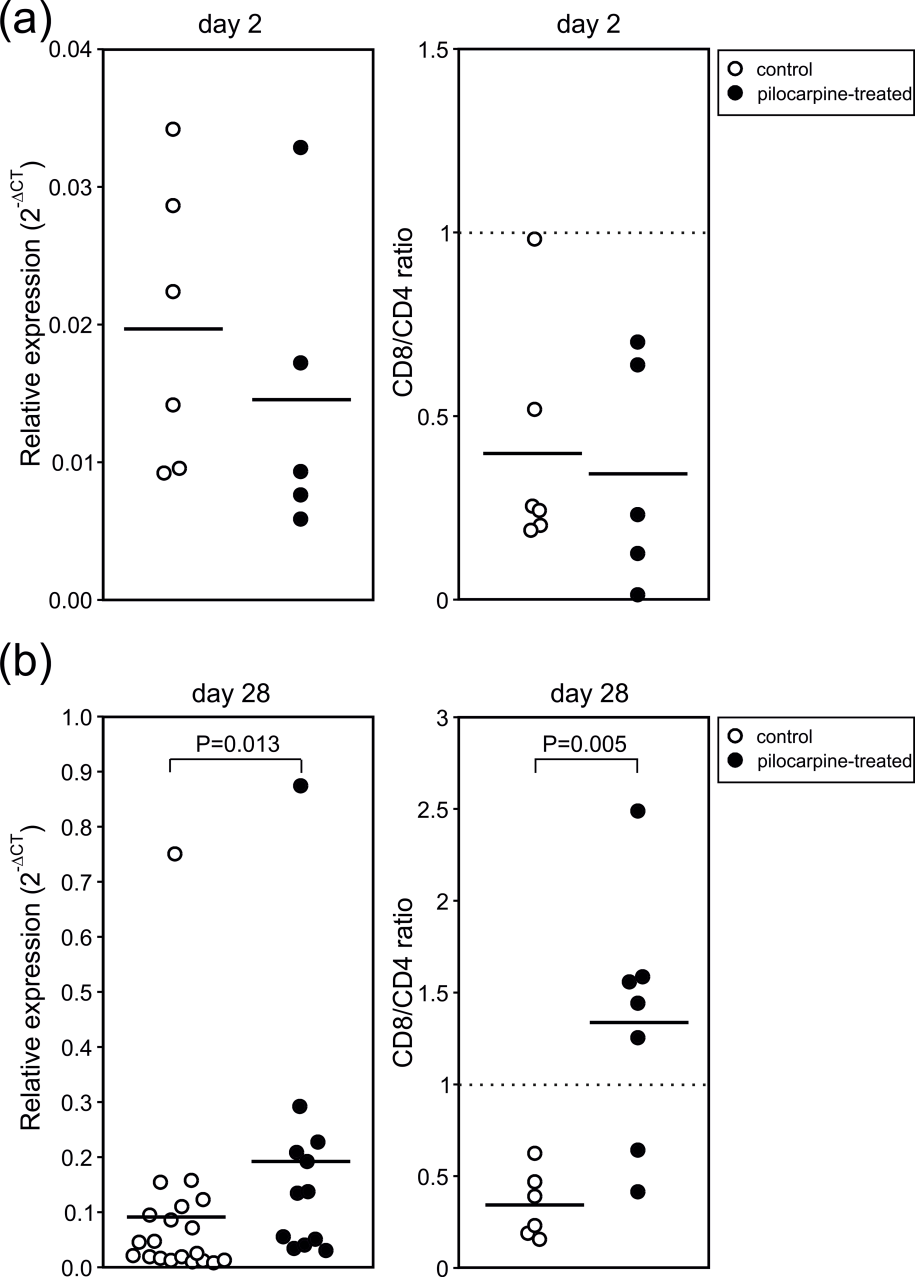
Quantitative PCR confirms the late response of CD3^+^ cells. Relative expressions of CD3g (normalized to Ptprc, 2^-ΔCT^) in pilocarpine-treated and control animals on day 2 **(a)** and on day 28 **(b)** after SE (left panels). The right panels show the CD8/CD4 ratios in both experimental groups at the respective time-points. Each symbol represents one animal. Horizontal lines indicate means, and P-values result from unpaired t-tests or U-tests (see text).

So far, we here described a biphasic immune response in the hippocampus with an immediate and transient innate response followed by a delayed presence of T-lymphocytes around day 28 after SE. This orchestrated immunological sequela suggests an adaptive immune response associated with the pathogenesis of epilepsy. Since our observations on the early response following pilocarpine-induced status epilepticus are consistent with previous reports confirming hippocampal microglia activation [1,21,25,26], we here focused on the delayed T cell response and set out to confirm these data using quantitative reverse transcriptase polymerase chain reaction (qRT-PCR). Fig 5 presents the PCR data using the gene probe CD3g as a marker for T-lymphocytes (expressed relative to the gene probe Ptprc encoding CD45). While on day 2 after SE no significant changes of CD3g transcript abundance between pilocarpine-treated and control animals were present, we were able to reconcile our delayed T cell increase on day 28 after SE (pilocarpine-treated: 19.0±6.4%, n = 12 animals; control: 9.0±3.6%, n = 20 animals; P<0.05, U-test, Fig 5B). We took advantage of the qRT-PCR in order to discriminate between CD4^+^ and CD8^+^ T-lymphocytes. Using these gene probes, we found equal CD8/CD4 ratios between pilocarpine-treated and control animals on day 2 after SE, but importantly, the CD8/CD4 ratio significantly increased in pilocarpine-treated animals on day 28 after SE as compared to age-matched controls (pilocarpine-treated: 1.34±0.24, n = 7 animals; control: 0.34±0.07, n = 6 animals; P<0.01, t-test, Fig 5B). Hence, the delayed T cell response was confirmed using qRT-PCR and, in addition, we found that the underlying T cell population was predominantly CD8 positive.

**Fig 6:**
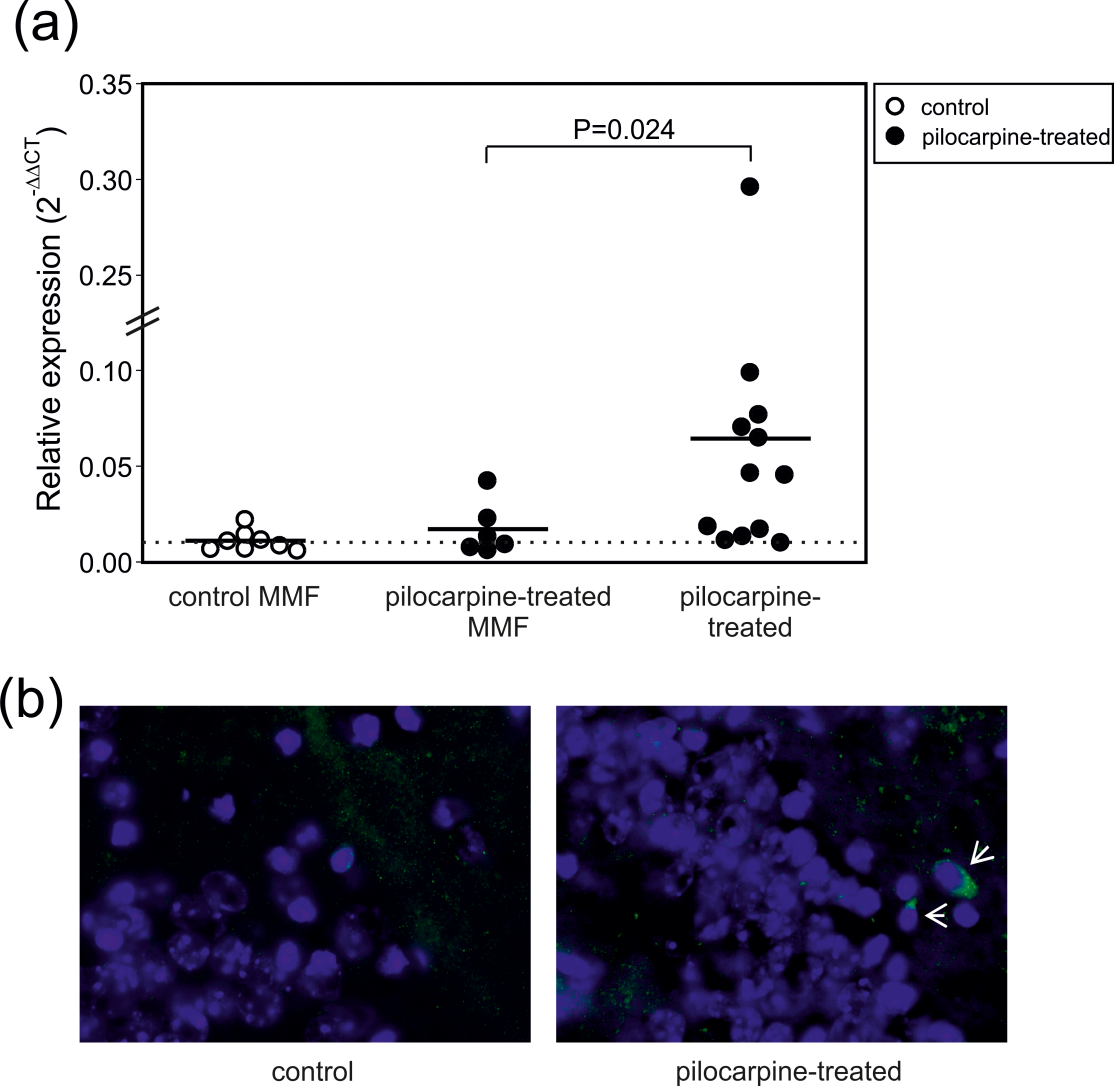
Mycophenolate mofetil prevents the delayed response of CD3^+^ cells. **(a)** Relative expression of CD3g (normalized to control, 2^-ΔCT^) in control animals treated with mycophenolate mofetil (MMF) as well as in pilocarpine-treated animals on day 28 after SE with or without MMF treatment. The relative expression shown results from the comparison to mice neither treated with pilocarpine nor with MMF. Note that MMF given on day 0–3 after SE had no effect in controls, but significantly prevented the increased CD3g abundance in pilocarpine-treated animals. Each symbol represents one animal. Horizontal lines indicate means, and P-values result from unpaired t-tests. **(b)** CD3 immunoreactivity in the dentate gyrus of a control (left) and a pilocarpine-treated animal (right). Individual CD3^+^ cells (green) are indicated by white arrows. Nuclei are stained with DAPI (blue color). Magnification 600x.

Next, we hypothesized that the delayed T cell response observed on day 28 post SE resulted from immunological processes elicited either during or shortly after status epilepticus. To test this, we administered the purine antagonist mycophenolate mofetil (MMF) to the animals immediately after SE termination with diazepam. Intriguingly, we observed that the CD3g transcript level which was elevated in pilocarpine-treated animals could be significantly restored to control values by this intervention (w/ MMF: 171±51%, n = 6; w/o MMF: 643±218%, n = 12; P<0.05, U-test, Fig 6A). On the other hand, MMF had no effect on CD3g transcript levels in control animals (open symbols in Fig 6A).

At last, we asked whether the delayed T cell response in the hippocampus observed via flow cytometry and qRT-PCR actually reflects T cell presence within the hippocampal parenchyma. We therefore performed immunohistochemistry of the dentate gyrus on thin sections of murine brains without and after pilocarpine-induced SE. CD3 immunoreactivity was infrequently found within the parenchyma, but 2/4 control mice showed CD3^+^ cells in the dentate gyrus (1.5±0.8 per mouse, 20 sections from 4 animals; Fig 6B). In contrast, in pilocarpine-treated mice, CD3^+^ cells were present in all animals tested (3.3±1.0 per mouse, 39 sections from 6 animals, P = 0.053, χ^2^ test; Fig 6B).

## Discussion

Inflammatory responses have increasingly been implicated in the pathogenesis of temporal lobe epilepsy [2]. However, the exact roles of the innate and adaptive immune system have not been delineated. Although mouse is the preferred animal species providing the opportunity of genetically modified tissue, low sensitivity to chemoconvulsants and high mortality hampered studies on experimental temporal lobe epilepsy in mice in earlier reports [14–17]. Moreover, C57BL/6 which is the most widely used inbred mouse strain, showed a minor cell loss in the dentate hilus compared to less commonly used strains [27]. Therefore, a ramping protocol with repeated injections of low doses of pilocarpine has been established to reduce mortality and increase the SE rate [18–20,27]. In our hands, consistent with these reports, roughly half of the pilocarpine-treated mice developed and survived SE.

In this study, we show a biphasic increase of CD45^+^ immune cells in the hippocampal brain parenchyma involving both, innate macrophages on the first four days and CD3^+^ T-lymphocytes representing the adaptive immune system around day 28 after pilocarpine-induced status epilepticus. Indeed, the hippocampal macrophages we found on days 1–4 showed an increased granularity and thus are indicative of an activated state. This activation of microglial cells has been shown to stimulate the expression of pro-inflammatory cytokines like IL-6, TNF-alpha and IL-1beta [28,29]. And the latter was implicated in negatively affecting the integrity of the blood brain barrier [3,4]. The remaining 30% of CD45^+^/CD11b^+^ cells were not investigated further, based on previous reports we assume that they might be infiltrating macrophages [30–33]. As for the early phase following pilocarpine-induced status epilepticus, our data are in line with previous reports and confirm that pilocarpine induces the activation of hippocampal microglia [1,21,25,26].

However, the transient nature of this first innate phase and its replacement by an adaptive response around day 28 is the first description of an immune kinetic associated with pilocarpine-induced epilepsy in the mouse. Even though activation of microglia and influx of CD3^+^ T cells was also described for the kainate model, the kinetics were different and may reflect the differences between both models [12]. But importantly, transfer of CD8^+^ T cells into β2-microgloblin-knockout mice significantly enhanced macrophage recruitment in the same model, suggesting a rather aggravating role of this cytotoxic T-cell population during epileptogenesis [34]. Furthermore, CD3^+^ cells with a CD4^+^ phenotype were observed after status epilepticus induced by a single, but higher dose of pilocarpine [35] probably leading to a more pronounced blood-brain barrier disruption that could be responsible for the early presence of CD3^+^ cells in the hippocampus.

In the present study, the delayed adaptive response observed via flow cytometry of hippocampal cell suspensions involved CD3^+^ T-lymphocytes and was confirmed twofold–via quantitative RT-PCR and immunohistochemistry of brain thin sections. The immunohistochemistry showed both, a perivascular location of CD3^+^ cells as well as a scattered distribution within the dentate gyrus and the Ammon's horn and thus demonstrates the infiltration of brain parenchyma with adaptive immune cells. It is a major finding of the present study that the quantitative RT-PCR data not only confirmed the delayed T-cell response, but was also able to demonstrate that the increase was attributable to an enhanced CD8^+^ T-cell population. While the pathophysiological role of CD8^+^ cells has already been suggested in the rodent kainate model [34], there is recent evidence on the pathogenic role of CD8^+^ cells in autoimmune anti-GABA-B-receptor encephalitis in humans [36], but intriguingly, also human temporal lobe epilepsy [37].

During the time span we analyzed, we neither detected a hippocampal increase in NK cells nor in B220^+^ B-lymphocytes following pilocarpine-induced status epilepticus. However, we did not yet monitor the development of central nervous system specific autoantibodies and thus cannot rule out at this stage that the B cell lineage is also involved in the pathogenesis of this particular animal model of temporal lobe epilepsy.

In summary, our data suggest that the pilocarpine-induced status epilepticus either leads to the infiltration of activated macrophages into the hippocampal brain parenchyma or to the activation of resident glial cells and their upregulation of macrophage markers. These innate immune cells are predicted to induce a pro-inflammatory immune response which on the one hand may attract more immune cells and on the other may affect the permeability properties of the blood brain barrier allowing T-lymphocytes to enter. We do not yet know whether brain specific effector or memory T cells proliferate elsewhere and are subsequently attracted into the hippocampus or whether proliferation occurs locally. However, the presence of numerous activated macrophages allows for the latter. Understanding in detail which immune mechanisms are involved in driving the epileptogenesis will help design novel therapeutical strategies with the potential of disease modification. Interestingly, ablating individual immune cells after kainate-induced status epilepticus hinted at neuroprotective roles for both, activated macrophages and lymphocytes [12]. In this sense, it is important to note that early and transient immunomodulatory intervention was able to prevent the delayed presence of T-lymphocytes. Thus, one of the imminent questions in need of resolving is the role of immunological memory during epileptogenesis.
